# Bacteria, Fungi, and Scalp Psoriasis: Understanding the Role of the Microbiome in Disease Severity

**DOI:** 10.3390/jcm13164846

**Published:** 2024-08-16

**Authors:** Jin-Young Choi, Hyunseong Kim, Kyung-Hyun Min, Woo-Hyun Song, Dong-Soo Yu, Minho Lee, Young-Bok Lee

**Affiliations:** 1 Department of Dermatology, College of Medicine, The Catholic University of Korea, Seoul 06591, Republic of Korea; 2 Department of Life Science, Dongguk University—Seoul, Goyang 10326, Republic of Korea; 3 Department of Tropical Medicine, Institute of Tropical Medicine, Yonsei University College of Medicine, Seoul 03722, Republic of Korea; 4 Department of Dermatology, Uijeongbu St. Mary’s Hospital, College of Medicine, The Catholic University of Korea, Uijeongbu 07345, Republic of Korea

**Keywords:** mycobiome, microbiome, psoriasis, scalp psoriasis, fungus, bacteria

## Abstract

**Background:** Psoriasis is a chronic skin condition affected by genetic and environmental factors. Changes in the skin microbiome may affect the immune system and skin barrier functions, thereby contributing to the development and progression of psoriasis. The scalp, which is a common site for psoriasis, is often resistant to therapy. Although several studies have investigated the scalp microbiome, analyses focusing on both bacteria and fungi remain scarce. **Methods:** We examined the scalp microbiomes of 11 patients with psoriasis complicated with scalp lesions and categorized them according to their Psoriasis Area Severity Index (PASI) scores. The bacterial and fungal data were analyzed using QIIME2 pipeline version 2021.04 and the UNITE database version 8.3, respectively. **Results:** The Shannon indices for mild (2 patients), moderate (4 patients), and severe (5 patients) groups were 0.97, 1.38, and 1.88, respectively. A significant correlation was observed between increased mycobiome diversity and disease severity (*p* = 4.07 × 10^−5^, Spearman’s correlation: 0.9269). Compared with the mild and moderate groups, the severe group exhibited a higher abundance of *Malassezia globosa*. *Pseudomonas* and *Staphylococcus* were, respectively, more prevalent in the moderate and severe groups than in the mild group. **Conclusions:** This study highlights the potential role of increased fungal diversity and specific microbial compositions in the severity of scalp psoriasis, suggesting a possible avenue for targeted therapeutic interventions.

## 1. Introduction

Psoriasis is a chronic inflammatory skin disease affecting 1–3% of the global population [1,2]. It is characterized by recurrent and persistent scaly erythematous plaques that frequently appear on the extensor skin and scalp. Genetic predispositions, environmental factors, and epigenetic modifications aggravate this disease [3,4]. For decades, the roles of skin and gut microbiota have been highlighted in psoriasis pathophysiology [5]. The skin microbiota includes fungi, bacteria, viruses, and mites that continuously interact with the host skin [6,7].

The skin microbiome of patients with psoriasis differs from that of healthy individuals. Altered abundance and diverse microbial populations have been observed in psoriatic lesions compared with those in non-lesional skin or skin from healthy controls [8,9]. For instance, the abundance of *Staphylococcus* increases and that of *Propionibacterium* decreases in psoriatic skin [10]. This microbial imbalance or dysbiosis may elicit characteristic inflammatory responses against psoriasis by promoting pro-inflammatory reactions, undermining skin barrier functions, or inducing autoimmunity via molecular mimicry [11,12].

Fungi, notably *Malassezia*, play a role in psoriasis [11], which has been supported by the observation that the topical application of *Malassezia* suspensions can induce psoriatic plaque formation [13]. This hypothesis is supported by the effectiveness of antifungal treatments in managing psoriasis [14,15]. Recently, the advent of biological therapeutics has made it possible to achieve the near clearance of psoriasis. However, psoriasis of the scalp, shins, and nails tends to persist despite the treatment [16]. Targeted monoclonal antibodies can improve the symptoms and dysbiosis of the skin microbiome in atopic dermatitis [17]. Conversely, Koike et al. found that the skin mycobiome of a patient with psoriasis was stable during biologic therapy [18].

The scalp is a common site of psoriasis in approximately 80% of the total number of patients [19]. Scalp psoriasis negatively affects quality of life owing to its visibility, and the lack of effective and standardized treatment strategies makes it worse [20]. Although several studies have investigated the scalp microbiome, analyses focusing on both bacteria and fungi remain scarce. In this study, a total of 11 patients with varying severity of scalp psoriasis were enrolled: 2 patients with mild scalp psoriasis, 4 patients with moderate scalp psoriasis and 5 patients with severe scalp psoriasis. Their skin metagenome was sequenced, and their bacterial and fungal profiles were compared across the different severity levels. By better understanding the microbiome associated with scalp psoriasis, we aim to gain insights into psoriasis pathophysiology and explore new strategies for treating recalcitrant scalp psoriasis.

## 2. Materials and Methods

### 2.1. Study Subjects and Sample Collection

We enrolled 11 patients with scalp psoriasis who had been diagnosed with psoriasis and had received treatment in the Dermatology Department of Uijeongbu St. Mary’s Hospital from September 2020 to November 2020. This study was performed in autumn in Korea, which is characterized by relatively stable weather conditions. Scalp microbiome samples (11) were collected twice from each patient from the same room of the hospital by the same dermatologist. Sample nomenclature was arrayed sequentially from S01 to S11, according to the order in which they were collected. “B” denotes bacteria, whereas “F” denotes fungi. Written informed consent was obtained from all patients, and the study was approved by the institutional review board of Uijeongbu St. Mary’s Hospital (UC20TESI0116). After taking photographs of the whole body, including the scalp, to calculate the Psoriasis Area Severity Index (PASI) score, samples of psoriatic plaques on the patients’ scalps were obtained by swabbing the skin with a cotton swab, which was deposited into a sterile tube. Data on the patients’ sex, age, current treatment, and severity were collected, and samples were stored in the Catholic Biobank.

### 2.2. Definition of PASI and Scalp PASI

To measure the severity of psoriasis, the PASI was calculated [21]. This index was developed in 1978, and it evaluates the degree of thickness, erythema, and scaling of the affected skin in four different body regions as follows: the head and neck, the upper limbs, the trunk, and the lower limbs. The subtotal of each body site is multiplied by the proportion of the body surface area to obtain a total score ranging from 0 to 72. The scalp PASI is a regional score that ranges from 0 to 7.2. The PASI was considered a continuous variable and was categorized into three groups based on the European recommendations for mild, moderate, and severe scalp psoriasis [22]. These recommendations consider the scalp area affected by psoriatic plaques and psoriasis features such as erythema, scaling, thickness, and pruritus [19].

### 2.3. DNA Extraction

To isolate bacterial and fungal DNA from the samples collected, the QIAamp DNA Mini spin column (Qiagen Co., Hilden, Germany) was used. The detailed method can be found elsewhere [23].

### 2.4. Fungal Identification by Internal Transcribed Spacer (ITS) Sequencing Analysis

ITS sequences were processed using the quantitative insights into microbial ecology (QIIME2) pipeline 2021.04. The sequences were demultiplexed and imported into QIIME2. Reads were trimmed from the 3′ end to achieve a read length of 220 bases, followed by filtration and deionization using DADA2 (q2-dada2 version 2021.4.0, DADA2 1.8) with a minimum quality score of 30. The UNITE database (version 8.3, 2021.05.10) was used as a reference database for taxonomic classification.

Community-level analyses, including the measurements of alpha and beta diversities, were performed using the BIOM table. To assess species evenness, Shannon’s and Simpson’s indices were calculated, both of which increased as the distribution of the sample species became more uniform. Principal coordinate analysis was performed using both Bray–Curtis dissimilarity and Weighted UniFrac distance metrics, which consider both species abundance and presence or absence [24,25,26].

### 2.5. Bacterial Identification

The V2–4 and V6–9 regions of 16S rDNA were sequenced using the Ion S5™ XL System (Thermo Scientific, Waltham, MA, USA) and primer set (Ion 16s Metagenomics kit, Thermo Scientific) [27]. The detailed method can be found in our previous study [23].

### 2.6. Prediction of Functional Profiling

The functional analysis of ITS data was performed using Phylogenetic Investigation of Communities by Reconstruction of Unobserved States 2 (PICRUSt2) v.2.2.0-b, which predicts functional abundances. A Kyoto Encyclopedia of Genes and Genomes ORTHOLOGY (KEGG ORTHOLOGY, KO) abundance file was generated using the PICRUSt2 script (picrust_pipeline.py) with the QIIME2 results.

### 2.7. Statistical Analysis

Relative abundance was calculated as the percentage of abundance occupied by each species within each sample. The Kruskal–Wallis and Wilcoxon rank-sum tests were used to determine whether there were significant differences in the relative taxonomic abundances, alpha diversities, and KO enrichment between the three severity groups (mild, moderate, and severe). Pearson correlation coefficients (r) and Spearman’s rank correlation (ρ) tests were used to compare the two groups as well as the scalp PASI and Shannon index.

## 3. Results

### 3.1. Demographics of the Patients with Scalp Psoriasis

The 11 patients with psoriasis who participated in this study suffered from well-controlled psoriasis, except that on the scalp; 11 scalp psoriasis samples were collected and analyzed (Table 1). The mean PASI score, including that for the scalp, for the 11 samples was 7.53 ± 6.29, and the patients were administered biologics (*n* = 7), immunomodulators (*n* = 3), or topical calcipotriol/betamethasone only (*n* = 1). The biologics used by the patients included secukinumab (*n* = 3), guselkumab (*n* = 1), and ustekinumab (*n* = 3). The mean scalp PASI of all samples was 1.87 ± 0.93, and 0.55 ± 0.35 for the mild, 1.4 ± 0.28 for the moderate, and 2.62 ± 0.54 for the severe group. Among them, one patient suffered from psoriatic plaques only on the scalp. The remaining patients with higher scalp PASI scores suffered from plaques on the whole body (the mean PASI was 1.95 ± 0.07, 8.13 ± 8.35, and 9 ± 5.47 for the mild, moderate, and severe scalp psoriasis groups, respectively).

### 3.2. Biodiversity According to the Severity of Scalp Psoriasis

#### 3.2.1. Fungal Biodiversity According to Scalp Psoriasis Severity

**Figure 1:**
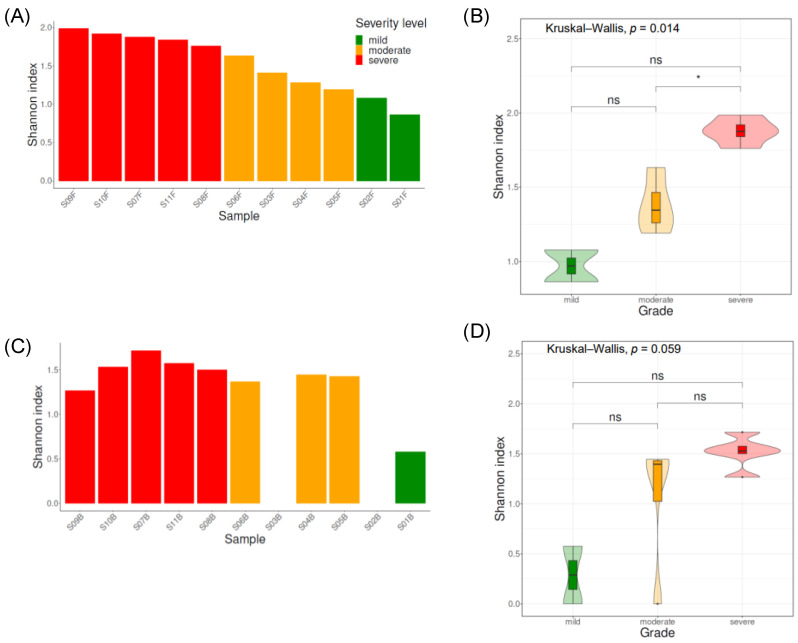
The fungal (**A**,**B**) and bacterial (**C**,**D**) Shannon indices according to the severity of the disease. *: *p* < 0.05 and ns: *p* > 0.05.

The alpha diversity of scalp psoriasis was examined according to the severity of the condition by calculating the Shannon index (Figure 1A,B). The mean Shannon indices were 0.97 ± 0.15, 1.38 ± 0.19, and 1.88 ± 0.08 in the mild, moderate, and severe groups, respectively. The score increased as the severity increased (*p* = 4.07 × 10^−5^, ρ = 0.9269).

#### 3.2.2. Bacterial Biodiversity According to Scalp Psoriasis Severity

Bacterial diversity was assessed by calculating the Shannon index (Figure 1C,D). The mean Shannon indices in the mild, moderate, and severe groups were 0.29 ± 0.41, 1.06 ± 0.71, and 1.52 ± 0.16, respectively. Similar to the trend observed in fungi, as the severity increased, diversity increased (r = 0.7337, *p* = 0.0102, and ρ = 0.7520, *p* = 0.0076).

**Figure 2:**
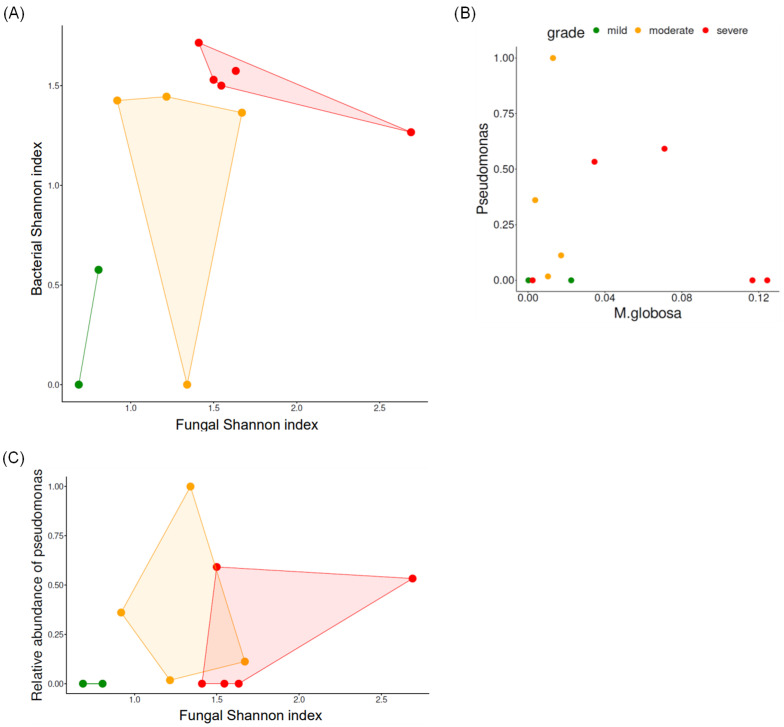
Correlation of the mycobiome with the bacterial microbiome. The principal component analysis plot showing the correlation between bacterial and fungal Shannon indices (**A**), the relative abundance of *Pseudomonas* and *M. globosa* (**B**), and the relative abundance of *Pseudomonas* and the fungal Shannon index (**C**).

Figure 2A depicts the Shannon index of the fungal and bacterial microbiomes. Samples from the severe group exhibited high biodiversity in either bacterial or fungal microbiomes.

### 3.3. Taxonomical Compositions of Each Severity Group

#### 3.3.1. Fungal Taxonomical Compositions of Each Severity Group

**Figure 3:**
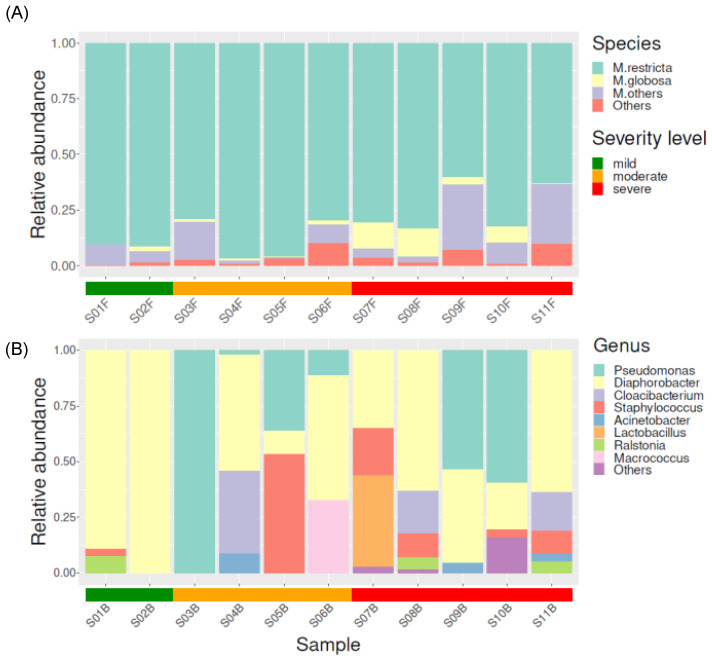
Taxonomic bar plot showing the relative abundance of *Malassezia* and other genera (**A**) and bacteria (**B**) in patients with scalp psoriasis, according to the severity of the condition.

**Figure 4:**
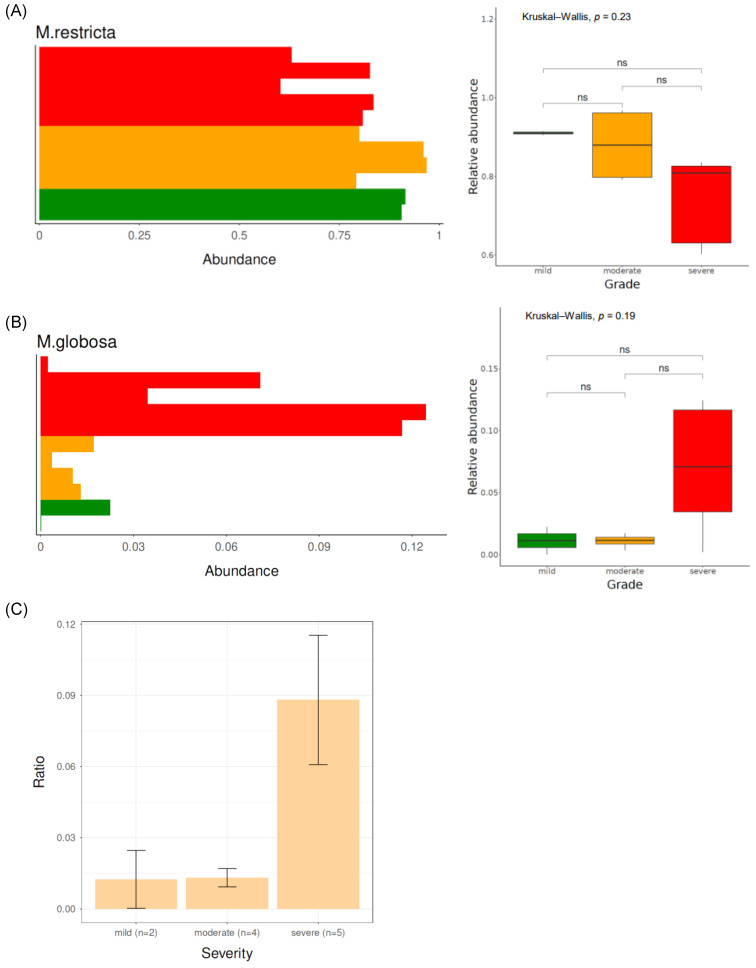
Relative abundance of *Malassezia restricta* (**A**) and *Malassezia globosa* (**B**) according to disease severity. The ratio of *Malassezia globosa* to *Malassezia restricta* in each severity group (**C**). The colors used in the figures are as follows: Green indicates the mild severity group, orange represents the moderate severity group, and red denotes the severe severity group. ns: *p* > 0.05.

Figure 3A shows the fungal microbiome composition at the genus level. *Malassezia* was the most dominant genus, followed by *Exophiala*, *Knufia*, *Aspergillus*, and *Cladosporium*. Among the *Malassezia* genus, the most abundant species was *Malassezia restricta*, followed by *Malassezia globosa*, *Malassezia arunalokei*, *Malassezia sympodialis*, and *Malassezia dermatis*. The relative abundance of *Malassezia restricta* in the mild group (0.91 ± 0.01) was higher than that in the severe group (0.74 ± 0.11) (*p* = 0.05) (Figure 4A). *Malassezia globosa* was more enriched in the severe group (relative abundance: 0.07 ± 0.05) than in the mild group (relative abundance: 0.01 ± 0.02) (*p* = 0.12) (Figure 4B). When examining the ratio of *Malassezia globosa* to *Malassezia restricta* among the different severity groups, the ratio was evidently most pronounced in the severe group, whereas it was lower in the mild and moderate groups (Figure 4C).

#### 3.3.2. Bacterial Taxonomical Compositions of Each Severity Group

**Figure 5:**
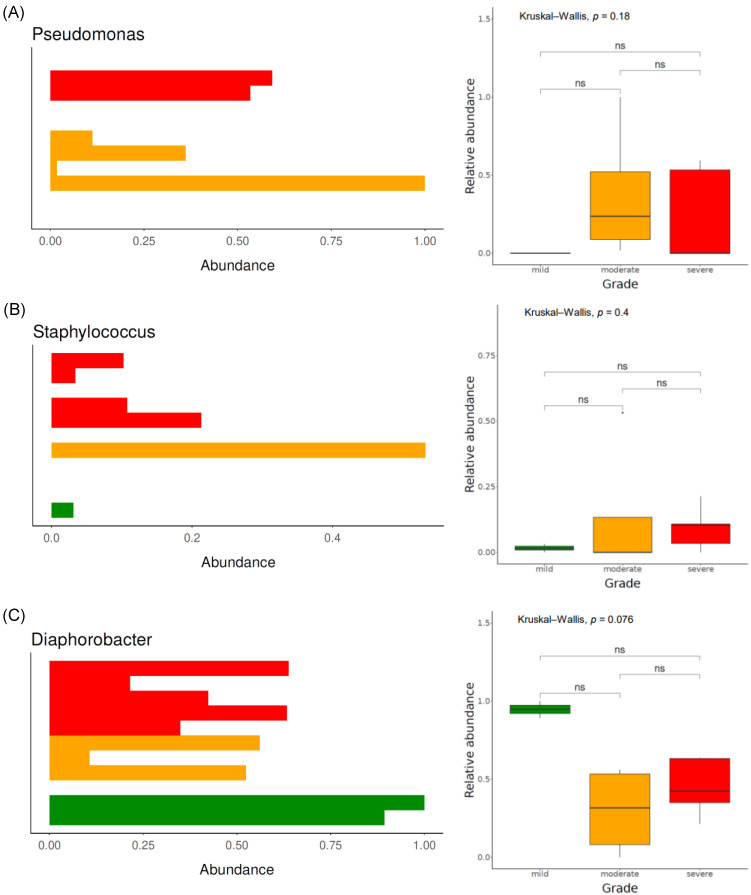
Relative abundance of the genera *Pseudomonas* (**A**), *Staphylococcus* (**B**), and *Diaphorobacter* (**C**) according to disease severity. The colors used in the figures are as follows: green indicates the mild severity group, orange represents the moderate severity group, and red denotes the severe severity group. ns: *p* > 0.05.

Figure 3B shows the bacterial microbiome composition at the genus level. *Diaphorobacter* was the most frequently detected genus, followed by *Pseudomonas*, *Staphylococcus*, *Cloacibacterium*, and *Acinetobacter*. Upon comparing the composition between each group, *Pseudomonas* was not detected in the mild group; however, it was abundantly present in both the moderate and severe groups. Similarly, *Staphylococcus* was scarcely present in the mild group; however, it was abundantly present in the moderate and severe groups. Conversely, *Diaphorobacter* exhibited the highest relative abundance in the mild group but was scarcely present in both the moderate and severe groups (Figure 5A–C).

#### 3.3.3. Functional Analysis

To predict fungal and bacterial gene functions in the metagenome, the PICRUSt2 algorithm was used. We did not find any statistically significant differences in terms of KO between the severity groups. None of the KOs reached statistical significance in the severity groups.

## 4. Discussion

The role of the microbiome in scalp psoriasis pathogenesis has received limited attention. A few studies have comprehensively analyzed the scalp microbiome in relation to disease severity. Herein, we examined the bacterial and fungal compositions of the scalp microbiome of patients with varying degrees of disease severity.

We found that microbial diversity increased with increasing disease severity; however, this increase was not statistically significant. This is consistent with a previous report indicating that psoriasis was associated with greater microbiome diversity as the disease became more severe [28]. Psoriatic skin exhibits increased microbial diversity compared with that exhibited by healthy skin [29]. Furthermore, our previous study on the skin microbiome revealed an increased relative abundance of *Pseudomonas* spp. in patients with severe scalp psoriasis [23]. Chang et al. showed that *Staphylococcus aureus* was closely correlated to psoriatic skin [10]. Similarly, samples from the severe group showed either an increased abundance of *Pseudomonas* or increased fungal alpha diversity (Figure 2). Bacteria and fungi probably interacted with each other within the scalp microbiome rather than existing independently.

*Malassezia* is associated with psoriasis, seborrheic dermatitis, atopic dermatitis, *Malassezia* folliculitis, and tinea versicolor [30]. It is lipophilic, which makes it inhabit seborrheic areas, such as the scalp [31]. It is particularly important to differentiate between scalp psoriasis and seborrheic dermatitis, both of which involve *Malassezia*. Both conditions present with scales and cause patients distress due to dandruff. Nonetheless, they can be differentiated by their typical areas of occurrence. Psoriasis usually exhibits characteristic plaques along the hairline and can manifest in regions beyond the scalp. The patients in this study were diagnosed with scalp psoriasis due to the presence of prominent psoriasis lesions on other parts of their bodies.

Herein, *Malassezia restricta* was abundantly present in psoriatic skin. Previous studies performed in Japan showed similar results, wherein *M. restricta* was the most frequently found species in psoriatic skin [32,33]. However, the most abundant species differed from those reported in other studies, wherein *M. furfur*, *M. globosa*, or *M. sympodialis* were abundantly present [31,34]. This discrepancy was attributed to differences in sampling methods, weather, and ethnicity.

The virulence of *Malassezia* in psoriasis pathogenesis partly stems from its lipase activity. Lipases and phospholipases produced by *Malassezia* damage the epidermal barrier and provoke an immune reaction, followed by inflammatory cytokine production by keratinocytes [35]. Among *Malassezia* spp., *M. globosa* exhibits the highest lipase activity and contains 14 genes that encode lipases [36]. One of the most studied genes is MgLip1, which is abundant on the human scalp, suggesting that the lipolytic enzyme encoded by this gene may contribute to *M. globosa* virulence [37]. These findings support the observation that *M. globosa* was more abundant in the severe group than in the other two groups. It hydrolyzes skin lipids into fatty acids for survival, according to a model of the role of *Malassezia* in dandruff and seborrheic dermatitis [38]. Diolein is used as a substrate to generate oleic acid, which can autonomously elicit dandruff [37,39]. Additionally, olive oil administration aggravates psoriasis by creating an imbalance between oleic and linoleic acid levels, thereby decreasing linoleic acid levels and increasing oleic acid levels in imiquimod- and olive oil-treated animals [40]. The measurement of oleic acid and *Malassezia* lipase levels in psoriatic skin may be a noteworthy topic for future research.

Interleukin (IL)-17 plays a key role in psoriasis pathogenesis [41]. Targeting IL-17 may improve the condition of patients with psoriasis [42]. IL-17 exerts antimicrobial effects, particularly on fungi [43,44]. People with a deficiency in the IL-17 pathway are more severely affected by fungal infections than those without any deficiency [45]. Sparber et al. found that *Malassezia* triggered IL-17 and IL-23 axes and aggravated skin inflammation [46]. The metabolites produced by *Malassezia* increased IL-36γ levels in human keratinocytes in vitro, thereby leading to IL-17 production [47]. The IL-17A gene single-nucleotide polymorphism and fungal growth are associated with psoriasis [48]. Although controversy exists regarding how *Malassezia* contributes to the disease, type 17 inflammation seems to be a bridge that connects *Malassezia* to psoriasis.

We integrated our findings on the fungal microbiome with those of our previous study results on the bacterial microbiome, which broadened our perspectives on the microbiome in scalp psoriasis. Although the present study showed similar results from a bacterial perspective to those of our previous study, the limited number of samples did not show statistically significant differences.

The homogeneous ethnicity of the participants is another strength of this study, although further studies including different ethnic groups are needed.

This study has a few limitations. First, the sample size was small, making the results statistically insignificant. Second, we did not include a control group with healthy skin. However, we categorized the groups according to the scalp PASI score and compared disease severity and scalp microbiome biodiversity. Third, no data were available on possible confounders, such as the use of antifungals and daily hair care routines, including shampooing. This was because the samples were collected for a short period in autumn to minimize the effect of weather changes on the skin microbiome. Thus, further studies with larger sample sizes are needed to confirm the present findings.

## 5. Conclusions

Although the study methodology had some limitations, such as a small sample size and the lack of a healthy control group, the present study revealed differences in the microbiome between patients with varying degrees of scalp psoriasis severity. *Malassezia* spp., especially *M. globosa*, might play a role in scalp psoriasis pathogenesis. Further studies are required to determine the effect of the microbiome on the disease and to strategize the applications of these findings to therapeutics.

## Figures and Tables

**Table 1:** Characteristics of the patients according to the severity of scalp psoriasis.

	Total (*n* = 11)	Mild Scalp Psoriasis (*n* = 2)	Moderate Scalp Psoriasis (*n* = 4)	Severe Scalp Psoriasis (*n* = 5)
Age, years (mean ± SD *)	41.36 ± 19.77	39.25 ± 23.44	41.6 ± 20.82	42.13 ± 19.61
Gender (Male/Female)	9:2	2:0	4:0	3:2
Scalp PASI (mean ± SD *)	1.87 ± 0.93	0.55 ± 0.35	1.4 ± 0.28	2.62 ± 0.54
PASI (mean ± SD *)	7.53 ± 6.29	1.95 ± 0.07	8.13 ± 8.35	9 ± 5.47
Current treatment				
Biologics	7	2	3	2
Cyclosporine	3	0	1	2
Topical steroid/calcipotriol	1	0	0	1

* SD, standard deviation.

## Data Availability

The data supporting the findings of this study are available from the corresponding author.
